# Timely integration of palliative care into oncology care for patients with bone metastases at the radiotherapy department: A pilot study on acceptability and feasibility

**DOI:** 10.1016/j.tipsro.2025.100317

**Published:** 2025-06-10

**Authors:** Anouk van Oss, Arianne Stoppelenburg, Ellen de Nijs, Rebecca van Jaarsveld, Carly S. Heipon, Natasja J.H. Raijmakers, Yvette M. van der Linden

**Affiliations:** a Center of Expertise for Palliative Care, Leiden University Medical Center, Leiden, the Netherlands; b Netherlands Comprehensive Cancer Organisation (IKNL), Utrecht, the Netherlands; c Department of Radiotherapy, Leiden University Medical Center, Leiden, the Netherlands

**Keywords:** Bone neoplasms, Palliative care, Patient acceptance of health care, Caregivers, Consultants, Feasibility studies

## Abstract

**Background and aim:**

Patients with bone metastases may have needs that extend beyond the management of pain by radiotherapy. Concurrent palliative care leads to improved quality of life, but is often introduced late. In this pilot study, we assessed the acceptability and feasibility of an introdu0000ctory conversation with a palliative care consultant at referral for palliative radiotherapy.

**Material and methods:**

Patients with bone metastases and their family caregivers were scheduled for an introductory conversation with a consultant from the hospital palliative care team. During this meeting, the potential benefits of integrating palliative care into their current or future care was discussed. Using statements on a 5-point Likert scale, patients and family caregivers independently evaluated the acceptability of the conversation, and consultants evaluated the feasibility.

**Results:**

Between December 2022 and March 2024, 48 patients were included in the study. Median age was 73 years, 63 % were male. Most patients (89 %) and family caregivers (96 %) appreciated the introductory conversation, were unaware of the existence of a palliative care team (60 %, 67 %, respectively), and would contact the team when having questions or concerns (77 %, 82 %). Some found the conversation confronting (17 %, 11 %), or felt it was too early in the illness trajectory (31 %, 26 %). Follow-up consultations were scheduled for 8 patients (17 %). Consultants were able to conduct the conversation as instructed (91 %), though 15 % indicated insufficient time for preparation.

**Conclusion:**

Introductory conversations about palliative care at referral for palliative radiotherapy appear both acceptable and feasible, and may enhance timely integration of palliative care into oncology care for patients with bone metastases.

## Highlights

• Patients with bone metastases and family accept timely palliative care introduction.
• Most patients are unaware of the existence of a hospital palliative care team.
• One third of patients wishes an in depth follow-up palliative care consultation.
• Introducing specialist palliative care at referral for radiotherapy is feasible.

## Introduction

Advanced cancer patients with bone metastases often experience significant bone pain and face an increased risk of pathological fractures and neurological complications [1]. Consequently, they may encounter difficulties with daily activities and a reduced quality of life (QoL) [2,3]. Palliative radiotherapy is a commonly used treatment for symptomatic bone metastases, achieving approximately a 60 % pain response rate [1,4]. Although symptom relief is important for improving QoL, patients with bone metastases and their family caregivers frequently have concerns extending beyond physical symptom management [[5], [6], [7]]. Advances in immunotherapy and targeted therapy aim to prolong life in patients with advanced cancer, but risks of overtreatment and potentially inappropriate end-of-life care may increase and illness trajectories become more uncertain, negatively impacting a patient’s QoL [7]. Therefore, timely discussion of goals and preferences for care and treatment is important.

Specialist palliative care (PC) provides support for physical symptoms, as well as for psychological, social, and spiritual needs [8]. Maintaining autonomy, ensuring access to information, enhancing effective communication between patients and clinicians, and incorporating advance care planning are essential parts of PC [8,9]. In hospitals in the Netherlands, specialist PC is provided by palliative care consultation teams (PCCTs). Since 2017, each hospital, caring for oncological patients, should have a PCCT [10,11]. International evidence shows that early integration of specialist PC into standard oncology care is a highly effective approach to further improve care and QoL for patients with advanced cancer. In a landmark study by Temel in 2010 [12], involving patients with metastatic non-small cell lung cancer, it was demonstrated that early PC leads to reduced depression and symptom burden, improved QoL, less potentially inappropriate end-of-life care, and prolonged survival. These positive results have been confirmed in two *meta*-analyses on early integration of PC into oncology care [13,14]. PC can be delivered concurrent with tumour-directed treatment, supporting patients and their family caregivers early in the illness trajectory [15].

Despite the known benefits, early referrals to PCCTs are currently limited, as misconceptions persist regarding PC being synonymous with end-of-life care [[16], [17], [18], [19]]. Other barriers to early integration of PC include lack of time during consultations of oncology clinicians, lack of knowledge about PC services, focus on the physical aspects of the disease, and low consensus between healthcare professionals on referral indicators [16,17,20,21]. Although not every patient with bone metastases may require referral to a PCCT, introducing the benefits of timely PC into their current or future care may enhance patient awareness and empowerment [22].

Determining the optimal timing for integrating PC can be challenging in patients with bone metastases, due to a variety of illness trajectories, symptoms, and wishes and needs of patients and families [7]. Several models have been introduced to improve timely integration of PC into oncology care: time-based, needs-based, and trigger-based [23,24]. Time-based criteria (disease stage, prognosis) and needs-based criteria (physical symptoms, performance status, psychological distress, end-of-life care planning), as proposed in the literature, present certain challenges [25]. Prognosis and illness trajectories are often poorly predictable, and identifying needs requires in-depth screening and is vulnerable to subjective interpretation of screening tools [24]. A trigger-based approach is based on predefined criteria and pathways streamlining the referral process [18,23]. For patients with bone metastases, an indication for palliative radiotherapy may serve as a clearly defined trigger to timely initiate PC.

In this pilot study, we assessed the acceptability and feasibility of timely integration of PC into oncology care for advanced cancer patients with bone metastases at the Radiotherapy Department. Our aim was to explore patient, family caregiver, and PC consultant views on an introductory conversation about the potential benefits of PC into current oncological care.

## Materials and methods

### Study design and setting

This single-centre, single-arm pilot study evaluated the acceptability and feasibility of an introductory conversation with consultants of the PCCT for patients with bone metastases at the Radiotherapy Department of the Leiden University Medical Center (LUMC). Patients from the LUMC and surrounding hospitals can be referred for palliative radiotherapy at the LUMC. The PCCT at the LUMC, established in 2012, consists of nurse practitioners and physicians specialized in palliative care, and works closely together with all departments that care for patients with incurable diseases. The PCCT provides multidimensional support and advises on medical, psychological, social and spiritual issues. The LUMC Medical Research Ethics Committee declared the study exempt from the Medical Research Involving Human Subjects Act (WMO; No. N21.136) The CONSORT checklist, including the extension for pilot and feasibility trials, was used for reporting [26].

### Participants

Patients eligible for this pilot study were adults (≥18 years), referred to the LUMC for palliative radiotherapy on bone metastases, and their family caregivers. For logistical reasons, this pilot study was able to schedule one or two patients per week for an introductory conversation with a PC consultant. We aimed to enrol 50 patients.

Based on the PCCT’s availability, eligible patients were scheduled for an appointment with a PC consultant on the same day as their visit with the radiation oncologist. Appointments at the Radiotherapy Department were commonly scheduled at short notice. Patients were informed about having an additional appointment with a nurse practitioner, but they were not provided with details regarding the nature of the conversation beforehand. All patients who had an introductory conversation with a PC consultant were invited to give informed consent to take part in this study. Patients who did not provide written informed consent were excluded from this study.

### Intervention

The PC consultants, all nurse practitioners, co-designed the content of the introductory conversation. A conversation guide for the introductory conversation was developed for standardisation of the intervention. This guide included the following topics: practical information about the conversation, introduction of the PCCT and the concept of palliative care, discussion of the patient’s current symptoms, an invitation for a more extensive follow-up consultation, practical information about the follow-up, and an invitation to provide informed consent and to complete a questionnaire. At the end of the introductory conversation, patients received a leaflet with information on what PC entails, the benefits of PC, and details of the PCCT. When patients requested a follow-up consultation, they also received the Leiden Guide on Palliative Care, a conversation guide that includes a symptom rating scale and a question prompt list to help patients and their family caregivers prepare for future consultations [[27], [28], [29]].

### Outcomes

The introductory conversation was evaluated on acceptability, from a patient and family caregiver perspective, and on feasibility, from a PC consultant perspective. Therefore, two questionnaires were developed by the project team, using the indicators of acceptability and feasibility of nursing interventions formulated by Sidani and Braden (2011) [30]. The project team consists of palliative care specialists, including a post-doctoral researcher (AS), two nurse practitioners of the PCCT (EN and RJ), and a radiation oncologist (YL). Based on the descriptions of the indicators provided by Sidani and Braden (2011) [30], statements for each indicator were developed and discussed within the project team. Other nurse practitioners of the PCCT provided feedback on the statements before they were finalised.

Both the patient and family caregiver (if present) were asked to complete a questionnaire about the acceptability of the introductory conversation. Eight statements were developed to measure five indicators of acceptability: appropriateness, effectiveness, adherence, convenience, and risks or adverse reactions (Table 1). Only patients who gave informed consent for this study were given a paper questionnaire to complete and return immediately after the conversation, or to send back at a later date. The PC consultant completed a questionnaire about the feasibility of the introductory conversation. Seven statements were developed to measure five indicators of feasibility: fidelity, context, availability and quality of the interventionists, material resources, and training of the interventionists (Table 1). All statements were measured on a 5-point Likert-scale (ranging from ’strongly disagree’ to ’strongly agree’). Both questionnaires allowed patients, family caregivers, and PC consultants to add comments about the introductory conversation.

**Table 1:** Statements regarding acceptability and feasibility according to Sidani and Braden [30].

*Patients and family caregivers*				
**Outcome**	**Indicator** [30]	**Description of indicator** [30]	**Statement in questionnaire**	**Short statement**
Acceptability	Appropriateness	Perception of the extent to which the intervention is helpful	“I like that an introduction with a PC consultant was scheduled for me/my loved one.”	Appreciate introduction
			“I found the conversation with the PC consultant too early in my/my loved one's illness trajectory.”	Introduction too early in illness trajectory
	Effectiveness	Perceptions of the intervention’s overall reasonableness and suitability	“Through this introduction I know what the PCCT can do for me/my loved one.”	Now know what PCCT can do
			“Before this introduction I already knew that there is a PCCT in the hospital.”	Knew existence PCCT prior to introduction
	Adherence	Extent to which they are willing to follow or adhere to the intervention	“If I have any questions or concerns, I will contact the PCCT.”	Will contact PCCT with questions/concerns
			“I would like a follow-up consultation with the PC consultant.”	Would like follow-up consultation
	Convenience	Judgement of the intervention’s intrusiveness	“I find it confronting that I/we had a conversation with a PC consultant.”	Introduction was confronting
	Risks or adverse reactions	Level of severity of the intervention’s adverse reactions/side effects	“The conversation with the consultant made me feel sad, angry, or worried about the future.”	Introduction evoked an emotional response

*PC consultants*				

**Outcome**	**Indicator** [**30**]	**Description of indicator** [**30**]	**Statement in questionnaire**	**Short statement**

Feasibility	Fidelity	Application of the intervention in the selected dose and the selected mode	“I conducted the conversation according to the instructions (on content and procedure).”	Followed instructions
	Context	Physical and social environment	“There was a suitable room to conduct the conversation.”	Suitable room available
			“I had enough time for the conversation.”	Sufficient time for introduction
	Availability and quality of interventionists	Adequate number of interventionists, personal and professional qualities	“I felt competent to carry out the conversation.”	Felt competent
			“I had enough time to prepare for the conversation.”	Sufficient time to prepare
	Material resources	Availability and preparation	“There was enough leaflet material available.”	Materials available
	Training of interventionists	Challenges and effectiveness	“The instructions (on content and procedure) of the conversation are clear.”	Clear instructions

**Fig. 1:**
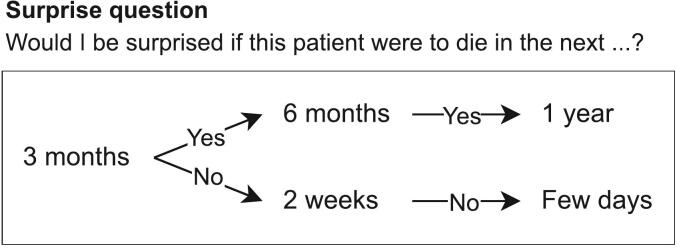
Adapted version of surprise question to estimate the expected survival by PC consultants.

Data on age, gender, living situation, performance status, primary tumour, radiotherapy schedule, radiotherapy site, systemic tumour-directed treatments, and survival were collected from the electronic medical record (EMR) for all patients who gave informed consent. Performance status was assessed using the Eastern Cooperative Oncology Group (ECOG) scale [31] as noted in the EMR, preferably measured by the treating physician. When information on performance status was not available in the EMR, the PC consultant assessed the ECOG score based on their clinical judgement. The PC consultant estimated the patient’s expected survival using an adapted version of the surprise question (Fig. 1) [32,33]. The patient’s actual survival period was collected three months after the last patient was included and categorized into < 3 months and ≥ 3 months after the introductory conversation.

In addition, the PC consultant noted the length of the introductory conversation and the presence and participation of a family caregiver during the conversation. Finally, it was assessed whether and when patients had had a follow-up consultation with the PCCT within six months following their introductory conversation.

### Data analysis

Descriptive statistics were used to describe sociodemographic and clinical characteristics, the duration of the introductory conversation, presence of a family caregiver during the conversation, timing and number of follow-up consultations, expected survival, and actual 3-month survival. Categorical variables were presented as observed counts and percentages, and continuous variables as median with range. The acceptability and feasibility of the introductory conversation were displayed in bar charts. For each statement, the 5-point Likert scale was also converted to a 3-point scale (‘strongly disagree/disagree’,’neutral’ and’agree/strongly agree’), and percentages were given. Open-ended comments from patients, family caregivers, and PC consultants aided the interpretation of the quantitative data. All data were analysed using IBM SPSS Statistics (version 29.0) and Rstudio (version 4.3.1).

## Results

**Fig. 2:**
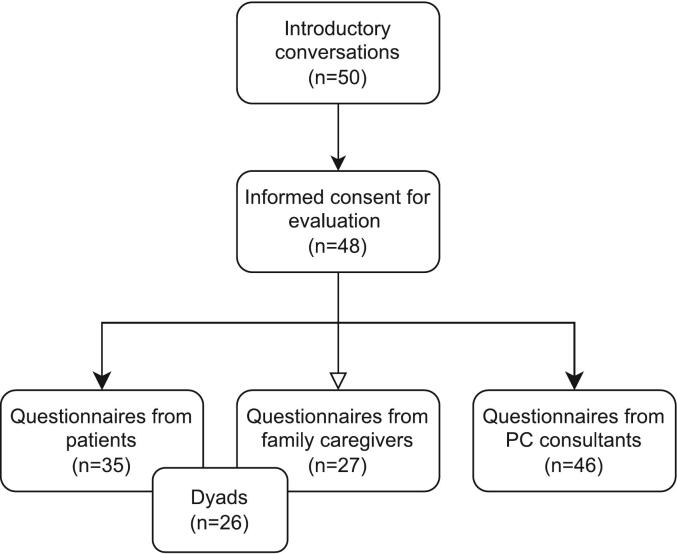
Flowchart of study population included in the medical data evaluation and analysis.

Between December 2022 and March 2024, a total of 50 patients with bone metastases attended an introductory conversation with a PC consultant at the Radiotherapy Department, and 48 patients gave informed consent for their medical data to be used for evaluation (Fig. 2). Data on acceptability was gathered from the patient and/or the family caregiver for 36 introductory conversations, of which 26 were assessed from both the patient and family caregiver perspective. For 12 introductory conversations no questionnaire was returned by either the patient or family caregiver.

The median age of patients was 73 years (range 24–91 years), and 30 (63 %) were male (Table 2). Most patients presented with bone metastases originating from prostate cancer (25 %) and were treated with a conventional radiotherapy schedule of 1x8 Gy (53 %). Half of the patients received systemic tumour-directed treatment at the time of the introductory conversation. The median duration of the introductory conversations was 15 min (range 5 – 20 min). A family caregiver was present for the majority of the introductory conversations (85 %), and most actively participated in the conversation (88 %). Most family caregivers were family members, including a partner, daughter or son, mother or father, or sister. In two introductory conversations an accompanying friend or professional caregiver was present, who are referred to as family caregivers.

**Table 2:** Baseline characteristics of 48 patients in pilot study.

	**All patients**	**Patients with a completed questionnaire on acceptability^1^**
	n = 48	n = 36
**Age**		
Median (range)	73 (24–91)	73 (24–91)
**Gender**, n(%)		
Male	30 (63)	22 (61)
Female	18 (38)	14 (39)
**Living situation**, n(%)		
Married/living together	30 (63)	25 (69)
Single/widow	12 (25)	9 (25)
Healthcare facility	2 (4)	1 (3)
Unknown	4 (8)	1 (3)
**Performance status^2^ (ECOG),** n(%)		
0	6 (13)	6 (17)
1	16 (33)	10 (28)
2	17 (35)	14 (39)
3	7 (15)	4 (11)
4	1 (2)	1 (3)
Unknown	1 (2)	1 (3)
**Primary tumour**, n(%)		
Prostate	12 (25)	8 (22)
Urologic	8 (17)	8 (22)
Breast	6 (13)	5 (14)
Lung	6 (13)	4 (11)
Gastrointestinal – colorectal	6 (13)	3 (8)
Other^3^	10 (21)	8 (22)
**RT schedule^4^**, n(%)		
1 × 8 Gy	29 (53)	25 (61)
2 × 8 Gy	13 (24)	7 (17)
5 × 4 Gy	9 (16)	6 (15)
10 × 3 Gy	2 (4)	2 (5)
No RT	2 (4)	1 (2)
**RT site^4^**, n(%)		
Vertebral column	18 (34)	15 (38)
Rib	8 (15)	7 (18)
Sacral	6 (11)	5 (13)
Pelvic bones	6 (11)	4 (10)
Femur	6 (11)	4 (10
Other	9 (17)	5 (13)
**Systemic tumour-directed treatment**, n(%)		
Yes	25 (52)	20 (56)
No	23 (48)	16 (44)
**Expected survival^5^**, n(%)		
>1 year	9 (19)	9 (25)
6 months – 1 year	23 (48)	16 (44)
3 months – 6 months	7 (15)	5 (14)
2 weeks – 3 months	5 (10)	3 (8)
<2 weeks	0 (0)	0 (0)
Unknown	4 (8)	3 (8)

Gy = Gray, ECOG = Eastern Cooperative Oncology Group.

^1^ Completed questionnaire on acceptability meaning at least one questionnaire has been returned from either the patient or the family caregiver.

^2^ Performance status assessed on a 0–5 scale, a higher grade indicating greater disability.

^3^ ‘Other’ category represents: hematologic, sarcoma, skin/melanoma, liver, thyroid, or adrenal gland.

^4^ Numbers exceed 48, because some patients received palliative radiotherapy on multiple bone metastases sites. ‘Other’ category represents: humerus, shoulder, skull, clavicle, sternum, or mandible.

^5^ Estimated by the PC consultants based on the surprise question [32,33].

### Acceptability of introductory conversation

**Fig. 3:**
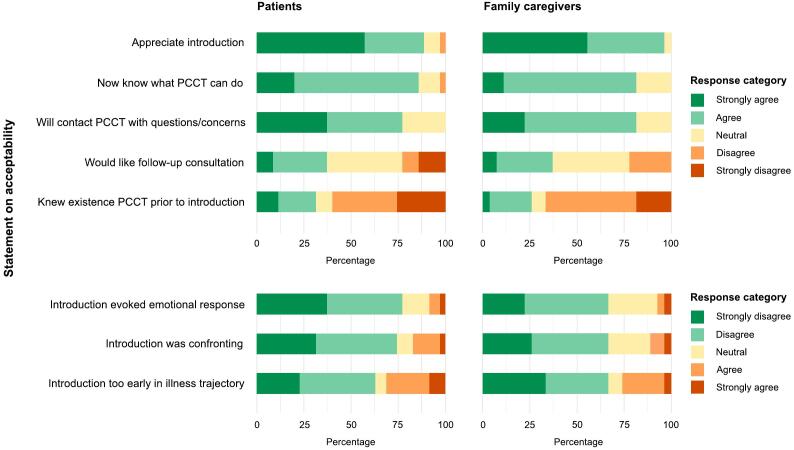
Acceptability of PC introductory conversation at Radiotherapy Department according to patients with bone metastases (n = 35) and their family caregivers (n = 27).

The majority of patients and family caregivers appreciated the introductory conversation (89 %, 96 % respectively) and did not find it too early in the illness trajectory (63 %, 67 %) (Fig. 3). Most patients and family caregivers were not aware of the existence of the PCCT (60 %, 67 %), but, after the introductory conversation, knew what the PCCT could do for them (86 %, 82 %), and would contact the PCCT if they had questions or concerns (77 %, 82 %). Some found the conversation confronting (17 %, 11 %), and felt sad or worried because of it (9 %, 7 %) (Appendix A: Table A1, Table A2).

### Follow-up

One third (37 %) of patients and family caregivers indicated that they would like a follow-up consultation. At the end of the study, 8 out of 48 patients (17 %) had had a follow-up consultation with a PC consultant. Main reasons for patients not wanting a follow-up consultation were: will ask questions to general practitioner or treating physician (9 times), already receives PC from another healthcare professional (7 times), no additional questions (7 times), or too early in the illness trajectory (4 times). The time between the introductory conversation and the follow-up consultation ranged from 5 days to 6 months. Although at the moment of the introductory conversation only 10 % of patients was expected to die within 3 months (Table 2), in reality, 15 out of 48 (31 %) patients died within 3 months of the introductory conversation. Of these patients, 3 had had a follow-up consultation with a PC consultant.

### Feasibility of the introductory conversation

**Fig. 4:**
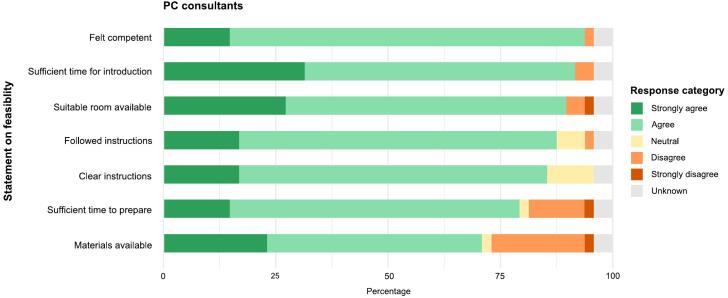
Feasibility of PC introductory conversation according to PC consultants (n = 46).

Five PC consultants held the introductory conversations, ranging from 5 to 23 times per consultant. They reported that the instructions were clear (89 %), they were able to conduct the consultation according to the instructions (91 %), and they felt competent to carry out the consultation (98 %) (Fig. 4). In 15 % of the cases there was not enough time to prepare for the consultation, and in 24 % of the cases the information materials were not complete (Appendix A: Table A3).

## Discussion

In this study we piloted clinical practice based timely integration of PC for patients with bone metastases, initiated when referred for palliative radiotherapy, incorporating experiences from patients, their family caregivers and PC consultants. The findings demonstrate that most patients and family caregivers appreciated the introduction to PC, were unaware of the existence of the PCCT and now know about the added value of the PCCT in their current or future care. From the PC consultants’ perspective it was feasible to have short introductory conversations at the Radiotherapy Department.

Some patients and family caregivers found the introductory conversation confronting or felt it came too early in the course of the illness. This may be explained by misconceptions about PC, associating PC with death, hopelessness, and end-of-life care, which often stem from the way it is communicated by healthcare professionals [[34], [35], [36]]. Addressing these misconceptions could enhance acceptability of timely integration of PC and help patients better recognize their own PC needs [37]. A previous study on early PC has shown that patients’ perceptions can shift following a consultation with a PC consultant, reframing PC as ongoing care focused on quality of life [34]. For a substantial number of patients in this pilot study, the introductory conversation with a PC consultant was their first interaction with PC, which may have caused the initial unsettlement with the term ‘palliative’ during the conversation. Following the introductory conversation, most patients recognized the added value of the PCCT and expressed a willingness to contact the team with any questions or concerns. Thus, when adequately explained, patients may be more willing to seek PC in the future.

We observed a lack of awareness of the existence of the PCCT in patients and family caregivers prior to the introductory conversation. This finding aligns with previous studies among patients with advanced cancer, which similarly reported limited awareness of PC services, potentially due to insufficient information sources [37,38]. Despite an increase in familiarity with the PCCT among healthcare professionals working in primary care teams, referrals for specialist PC remain initiated at a late stage in patients’ illness trajectories [21]. Barriers for healthcare professionals to introduce PC, including misconceptions and stigma about PC, lacking knowledge on what PC entails, concerns that using the term ‘palliative’ may take away hope, concerns of losing control of a patient’s care process, and uncertainty about the optimal timing, are complicating timely integration of PC for patients with bone metastases [17,18,[38], [39], [40]]. In the current study, consultants of the PCCT felt competent to conduct the introductory conversations about PC. However, when expanding the integration of PC into oncology care outside of this pilot study, the responsibility of informing patients about PC will likely shift to other healthcare professionals, who have to feel confident in taking on this role. Insights from this pilot study may enhance physicians’ and nurses’ confidence to start a conversation on benefits of PC, knowing patients appreciate timely introduction.

It is likely that not every patient requires specialist PC from a PCCT at the time of referral for palliative radiotherapy. A significant number of patients in this pilot study (19 %) said they would ask questions on PC to their general practitioner or treating physician. This finding highlights the importance of a mixed generalist – specialist PC model, in which all healthcare professionals who care for patients with a life-threatening disease are expected to integrate basic PC into their usual care [9,41]. PCCTs can be consulted to provide specialist PC, i.e., extra support for more complex problems. Healthcare professionals in primary care play an important role in recognizing patients’ PC needs. Despite the increasing integration of PC education and training into Dutch healthcare curricula, primary care providers still report insufficient education and knowledge in the PC domain [38,[42], [43], [44], [45]]. Improved education and training may take away barriers to introduce PC at a timely basis [38].

A Dutch nationwide study showed that the majority of patients who died with cancer in 2017, and for whom PC was initiated, received generalist PC only (88 %) [9]. Although the number of specialist PC consultations of hospital PCCTs has increased over the past decade, large differences in referral rates exist between PCCTs and referrals mostly do not occur until the last month of life [46]. A recent report of Dutch origin indicates that PCCTs are not as involved in the care of patients with advanced cancer as desired by PC consultants (2 % versus 33 %, respectively) [47]. Introducing PC when referring for palliative radiotherapy may prompt earlier identification of patients in need of specialist PC.

Although 37 % of the patients said they would like a follow-up consultation with the PCCT after the introductory conversation, 17 % had had a follow-up consultation. This difference may be explained by the fact that patients engaged in the introductory conversation on the same day as their appointment with the radiation oncologist. Their attention may have been primarily directed towards the treatment of their pain symptoms (i.e. radiotherapy treatment), which may have led to less consideration of the potential benefits of a follow-up consultation with the PCCT. In the absence of a follow-up consultation, the short introductory conversation may have prompted dialogue about PC with the patient’s general practitioner, treating physician, or family, or the scheduling of a follow-up PC consultation at the referring hospital [22]. Therefore, an introductory conversation about PC has the potential to enhance awareness of both patients and family caregivers, and reduce barriers to seek PC.

Some limitations of this pilot study need to be addressed. First, we did not collect (reasons for) non-recruitment systematically, resulting in a lack of information on recruitment rate. Second, there was some missing data on acceptability (27–44 %, patients and family caregivers respectively) and feasibility (4 %) of the introductory conversations. In some cases, patients and their family caregivers completed the questionnaire together, resulting in the submission of only one questionnaire per conversation. This might explain the high percentage of missing data in family caregivers. We assume that patients who returned a questionnaire about acceptability are representative of all patients who participated in an introductory conversation, given the comparable baseline characteristics (Table 2). However, this cannot be confirmed with certainty. Third, the acceptability questionnaire was developed without the involvement of patients, which potentially left out valuable statements to measure the acceptance of the introductory conversation. Lastly, the pilot study was conducted in a single academic hospital, limiting the generalizability of the findings to broader hospital settings. In particular, the feasibility of the introductory conversation may vary across different hospitals depending on the organizational structure and workflow.

In conclusion, there is considerable room for improvement in the timely integration of PC for patients with bone metastases. All healthcare professionals caring for patients with advanced cancer have a responsibility to introduce PC in a timely manner, taking into account the sensitivity of the term ‘palliative’. If implemented systematically into the hospital workflow, referral for palliative radiotherapy for bone metastases may serve as a trigger to initiate a conversation on PC. Future research is needed to evaluate the impact of this more systematic approach to timely integration of PC on QoL and satisfaction with care among patients with bone metastases and their family caregivers.

## Informed patient consent

The author(s) confirm that written informed consent has been obtained from the involved patient(s) or if appropriate from the parent, guardian, power of attorney of the involved patient(s); and, they have given approval for this information to be published in this case report (series).

## Declaration of competing interest

The authors declare that they have no known competing financial interests or personal relationships that could have appeared to influence the work reported in this paper.

## Figures and Tables

**Table A1:** Acceptability of introductory conversation according to patients (n = 35).

	*Patients (n = 35)* n (%)				
*Statements*	***Totally agree***	***Agree***	***Neutral***	***Disagree***	***Totally disagree***
I like that an introduction with a PC consultant was scheduled for me/my loved one.	20 (57.1)	11 (31.4)	3 (8.6)	0 (0.0)	1 (2.9)
I found the conversation with the PC consultant too early in my/my loved one's illness trajectory.	3 (8.6)	8 (22.9)	2 (5.7)	14 (40.0)	8 (22.9)
Through this introduction I know what the PCCT can do for me/my loved one.	7 (20.0)	23 (65.7)	4 (11.4)	1 (2.9)	0 (0.0)
Before this introduction I already knew that there is a PCCT in the hospital.	4 (11.4)	7 (20.0)	3 (8.6)	12 (34.3)	9 (25.7)
If I have any questions or concerns, I will contact the PCCT.	13 (37.1)	14 (40.0)	8 (22.9)	0 (0.0)	0 (0.0)
I would like a follow-up consultation with the PC consultant.	3 (8.6)	10 (28.6)	14 (40.0)	3 (8.6)	5 (14.3)
I find it confronting that I/we had a conversation with a PC consultant.	1 (2.9)	5 (14.3)	3 (8.6)	15 (42.9)	11 (31.4)
The conversation with the consultant made me feel sad, angry, or worried about the future.	1 (2.9)	2 (5.7)	5 (14.3)	14 (40.0)	13 (37.1)

**Table A2:** Acceptability of introductory conversation according to family caregivers (n = 27).

	*Family caregivers (n = 27)* n (%)				
*Statements*	***Totally agree***	***Agree***	***Neutral***	***Disagree***	***Totally disagree***
I like that an introduction with a PC consultant was scheduled for me/my loved one.	15 (55.6)	11 (40.7)	1 (3.7)	0 (0.0)	0 (0.0)
I found the conversation with the PC consultant too early in my/my loved one's illness trajectory.	1 (3.7)	6 (22.2)	2 (7.4)	9 (33.3)	9 (33.3)
Through this introduction I know what the PCCT can do for me/my loved one.	3 (11.1)	19 (70.4)	5 (18.5)	0 (0.0)	0 (0.0)
Before this introduction I already knew that there is a PCCT in the hospital.	1 (3.7)	6 (22.2)	2 (7.4)	13 (48.1)	5 (18.5)
If I have any questions or concerns, I will contact the PCCT.	6 (22.2)	16 (59.3)	5 (18.5)	0 (0.0)	0 (0.0)
I would like a follow-up consultation with the PC consultant.	2 (7.4)	8 (29.6)	11 (40.7)	6 (22.2)	0 (0.0)
I find it confronting that I/we had a conversation with a PC consultant.	1 (3.7)	2 (7.4)	6 (22.2)	11 (40.7)	7 (25.9)
The conversation with the consultant made me feel sad, angry, or worried about the future.	1 (3.7)	1 (3.7)	7 (25.9)	12 (44.4)	6 (22.2)

**Table A3:** Feasibility of introductory conversations according to PC consultants (n = 46).

	*PC consultants (n = 46)* n (%)				
*Statements*	***Totally agree***	***Agree***	***Neutral***	***Disagree***	***Totally disagree***
I conducted the conversation according to the instructions (on content and procedure).	8 (17.4)	34 (73.9)	3 (6.5)	1 (2.2)	0 (0.0)
There was a suitable room to conduct the conversation.	13 (28.3)	30 (65.2)	0 (0.0)	2 (4.3)	1 (2.2)
I had enough time for the conversation.	15 (32.6)	29 (63.0)	0 (0.0)	2 (4.3)	0 (0.0)
I felt competent to carry out the conversation.	7 (15.2)	38 (82.6)	0 (0.0)	1 (2.2)	0 (0.0)
I had enough time to prepare for the conversation.	7 (15.2)	31 (67.4)	1 (2.2)	6 (13.0)	1 (2.2)
There was enough leaflet material available.	11 (23.9)	23 (50.0)	1 (2.2)	10 (21.7)	1 (2.2)
The instructions (on content and procedure) of the conversation are clear.	8 (17.4)	33 (71.7)	5 (10.9)	0 (0.0)	0 (0.0)
